# Effects of chronic mild hyperoxia on retinal and choroidal blood flow and retinal function in the DBA/2J mouse model of glaucoma

**DOI:** 10.1371/journal.pone.0266192

**Published:** 2022-03-25

**Authors:** Eric R. Muir, Saurav B. Chandra, Divya Narayanan, Vincent Zhang, Ike Zhang, Zhao Jiang, Jeffrey W. Kiel, Timothy Q. Duong

**Affiliations:** 1 Department of Radiology, Stony Brook University, Stony Brook, NY, United States of America; 2 Research Imaging Institute, University of Texas Health San Antonio, San Antonio, TX, United States of America; 3 Department of Ophthalmology, University of Texas Health San Antonio, San Antonio, TX, United States of America; 4 Department of Radiology, Albert Einstein College of Medicine, Bronx, NY, United States of America; Transilvania University of Brasov: Universitatea Transilvania din Brasov, ROMANIA

## Abstract

**Purpose:**

To test the hypothesis that mild chronic hyperoxia treatment would improve retinal function despite a progressive decline in ocular blood flow in the DBA/2J mouse model of glaucoma.

**Materials and methods:**

DBA/2J mice were treated with chronic mild hyperoxia (30% O_2_) beginning at 4.5 months of age or were untreated by giving normal room air. Retinal and choroidal blood flow (RBF and ChBF, respectively) were measured at 4, 6, and 9 months of age by MRI. Blood flow was additionally measured under hypercapnia challenge (5% CO_2_ inhalation) to assess vascular reactivity. Intraocular pressure (IOP) was measured using a rebound tonometer at the same time points. Scotopic flash electroretinograms (ERGs) were recorded at 9 months of age.

**Results:**

Both ChBF and RBF were reduced and significantly affected by age (p < 0.01), but neither were significantly affected by O_2_-treatment (p > 0.05). ChBF significantly increased in response to hypercapnia (p < 0.01), which was also unaffected by O_2_-treatment. Significant effects of age (p < 0.001) and of the interaction of age with treatment (p = 0.028) were found on IOP. IOP significantly decreased in O_2_-treated mice at 6 months compared to 4 months of age (p < 0.001), while IOP trended to increase with age in untreated mice. The amplitude of the b-wave from ERG was significantly increased in O_2_-treated DBA/2J compared to the untreated mice (p = 0.012), while the a-wave and oscillatory potentials were not significantly affected (p > 0.05).

**Conclusion:**

This study investigated the effects of chronic mild hyperoxia on retinal function and on retinal and choroidal blood flow in a mouse model of glaucoma. Retinal function was improved in the O_2_-treated mice at late stage, despite a progressive decline of RBF and ChBF with age that was comparable to untreated mice.

## Introduction

Many glaucoma patients have elevated intraocular pressure (IOP), which is a major disease risk factor. Lowering IOP, by eye drops or surgery, is currently the only treatment for glaucoma [1]. However, in many patients, lowering IOP is inadequate to prevent vision loss [2, 3]. IOP is not the only factor involved in glaucoma [1, 2, 4]. Elevated IOP also reduces the ocular perfusion pressure (difference between arterial blood pressure and IOP), which might reduce blood flow to the retina, causing mild chronic blood flow deficits, leading to retinal ganglion cell dysfunction and degeneration [5, 6]. Vascular dysfunction and blood flow impairment by mechanisms other than elevated IOP are also associated with glaucoma, including systemic vascular abnormalities such as hypotension and vasospasm. These could impair the ocular circulations’ ability to maintain blood flow [4–6]. Furthermore, reduced retinal microvascular density occurs in experimental and human glaucoma, which could also lead to blood flow deficits [7–9].

We have previously shown that retinal and choroidal blood flow (RBF and ChBF, respectively) are reduced in the DBA/2J mouse model of glaucoma compared to age-matched normal control mice [10]. This suggests a progressive, chronic ischemic contribution to glaucomatous optic neuropathy and visual dysfunction in DBA/2J mice [11–17]. We have also found that lowering IOP using topical dorzolamide had the greatest effect to increase RBF and ChBF in late-stage DBA/2J mice, suggesting that IOP-lowering treatment may also help to normalize ocular blood flow [18]. The evidence that reducing IOP increases ocular blood flow suggests there are multiple hemodynamic effects involved in the vascular hypothesis of glaucoma.

Many studies have shown possible hypoxia-induced effects within the optic nerve head (ONH) and retina which could be caused by decreased blood flow and ocular hypertension [5, 19–22]. The endothelin-1 model of chronic optic nerve ischemia has shown that chronic ocular blood flow deficits can cause optic neuropathy similar to glaucoma [20]. Consistent with blood flow abnormality in glaucoma, hypoxic immunohistochemical markers have been found in the retina and optic nerve in clinical and experimental glaucoma [23]. The optic nerve head is susceptible to metabolic stress, with mitochondrial dysfunction and energy failure occurring here in glaucoma [24]. Improving oxygenation of the retina may thus provide an alternative strategy to prevent glaucomatous progression.

The purpose of this study was to longitudinally investigate the effects of mild hyperoxia treatment on the progressive deficits of ocular blood flow and visual function in the DBA/2J mouse model of glaucoma. DBA/2J mice were chronically housed in 30% oxygen from early to late-stages of glaucoma and were compared to untreated DBA/2J mice housed in normal room air. Retinal and choroidal blood flow were measured with magnetic resonance imaging (MRI), and retinal function was assessed by electroretinography (ERG). We hypothesized that DBA/2J mice treated with chronic mild hyperoxia would have improved visual function despite a progressive decline in ocular blood flow, supporting the hypothesis that mild, chronic hypoxic ischemia has a pathogenic role in glaucoma.

## Materials and methods

### Animal preparation

This study was carried out in accordance with the recommendations in the Guide for the Care and Use of Laboratory Animals of the National Institutes of Health. The protocol was approved by the Institutional Animal Care and Use Committee at the University of Texas Health Science Center at San Antonio (# 10046x). The DBA/2J mouse model of glaucoma (male, n = 21 total; Jackson Laboratory, Bar Harbor, ME) was studied at 4, 6, and 9 months of age. Studies were generally performed longitudinally on the same animals. However, two untreated mice died (or were euthanized due to injury from fighting), one by 6 months and one by 9 months, and two additional untreated mice were studied only at 9-months; additionally, one O_2_-treated mouse was found to have a collapsed left globe on the 6-month MRI, and while all data at 9 months appeared normal, all types of data from 6- and 9-months from the left eye were discarded. Animals were housed in our institutional animal facilities in typical mouse cages under a 12hr/12hr light/dark cycle and received a standard rodent diet (Teklad LM-485, Harlan, Houston, TX).

Animals were either treated with chronic mild hyperoxia (30% O_2_) beginning at 4.5 months of age or were untreated in normal room air in the animal facilities. Initial blood flow and IOP measurements at 4 months of age were made prior to initiating the hyperoxia treatment. Hyperoxia treated mice were placed in a sealed, feedback-regulated oxygen chamber (ProOx 110 with A-Chamber, BioSpherix, Parish, NY), in which oxygen in-flow was regulated to maintain the oxygen level in the chamber at 30% O_2_. Fans circulated the air within the chamber, and several small holes on three sides of the chamber allowed a constant flow of fresh gas mixture through the chamber. Two cages of mice were placed side-by-side in the chamber at once. Carbon dioxide levels in the chamber were occasionally tested using a capnometer (V9004 Capnograph, Surgivet, Waukesha, WI) and were always below the lower limit of detection of the capnometer (~0.5%). A hygrometer was used to monitor humidity in the chamber, and during more humid months, trays of silica desiccant beads were placed in the chamber as needed to maintain relative humidity under 60%. Mice were removed from the oxygen chamber and housed in room air for 24 hrs preceding experimental procedures at 6 and 9 months of age, so that studies could be conducted under normal room air conditions for all groups.

### MRI measurements of blood flow

MRI was used to measure the retinal blood flow and choroidal blood flow in DBA/2J mice aged 4-months, 6-months, and 9-months. Three MRI studies were discarded due to substantial artifacts from motion or improper imaging coil placement (one each from 4 mo O_2_, 9 mo air, and 9 mo O_2_ groups). For MRI, animals were placed in a holder with ear and tooth bars to minimize head motion. Mice spontaneously breathed room air with ~1.5% isoflurane for anesthesia. Respiratory rate was monitored, and slight adjustments made to the level of anesthesia to keep the respiratory rate in a target range of 80–120 breaths per minute. Animal temperature was monitored and maintained at 37°C by a circulating warm water pad on which the mouse laid for the duration of the experiment. MRI blood flow measurements were made with the animal breathing room air and under hypercapnia challenge (breathing air mixed with 5% CO_2_) to assess vascular regulation.

MRI was performed on a 7 Tesla magnet (Biospec, Bruker, Billerica, MA) with a 1500 mT/m BGA6S gradient insert. A custom eye coil was used for imaging the left eye (diameter = 6 mm) and a circular coil (diameter = 8 mm) was placed at the heart for continuous arterial spin labeling (ASL) [25, 26]. A gradient-echo, echo planar imaging sequence was used with a 6x6 mm^2^ field of view and a 144x144 matrix (42x42 μm^2^ resolution) reconstructed with zero-filled interpolation to 256x256. A single 400 μm thick slice was acquired with the imaging plane positioned through the optic nerve and bisecting the eye into superior and inferior segments. Other scan parameters were a 3.0 s repetition time, 9.7 ms echo time, two shots, partial Fourier factor of 0.76. Continuous ASL utilized a 2.6 s radiofrequency pulse to the labeling coil in the presence of 20 mT/m gradient, and a post-labeling delay of 350 ms was used. The sign of the frequency offset of the labeling pulse was switched for non-labeled images to compensate for magnetization transfer. Paired labeled and non-labeled images were acquired in an interleaved fashion. Two 10 min scans were run on each mouse, with the animal breathing air for the first 5 min and breathing the hypercapnic mixture during the final 5 min of each scan. Images for the first minute after switching to hypercapnia were excluded from analysis to allow time for the blood flow response.

MRI data were analyzed with custom software made in Matlab (MathWorks Inc, Natick, MA) [27]. Time-series data were first corrected for global motion using Statistical Parametric Mapping software (SPM12, Wellcome Centre for Neuroimaging, University College London, UK) [28]. A semi-automated process was then used to linearize the retina, correct retinal motion, and conduct an automated profile analysis [27]. The blood flow (ml/g/min) was calculated from the signal intensities of labeled and non-labeled images as previously reported [26]. Blood flow profiles were averaged along the length of the retina, and the peak RBF and ChBF were recorded. Blood flow values during periods breathing room air and during hypercapnia were calculated separately.

### Intraocular pressure measurements

IOP was measured with a rebound tonometer (Icare Tonolab, Helsinki, Finland) [29] on both eyes of mice directly before MRI procedures. IOP was additionally measured on four 4-month old mice which did not undergo MRI, while two mice in the untreated group did not have IOP measured at 4 months. Measurements were made under ~1.5% isoflurane with animals warmed using a heating pad. An average of 6 readings were taken per eye.

### Electroretinogram

Scotopic ERGs were recorded in DBA/2J mice at 9 months of age. Following over-night dark adaptation, animals were initially anesthetized with isoflurane and maintained at 1.5% delivered through a nose cone. Pupils were dilated with 1% tropicamide and 2.5% phenylephrine. Eyes were anesthetized using 0.5% proparacaine and lubricated with 0.5% carboxymethylcellulose. Body temperature was maintained at 37°C using a thermostatically controlled heating pad throughout the recordings. Binocular recordings were obtained using mouse ERG contact lens electrodes (LKC technologies, Gaithersburg, MD) with needle electrodes (OcuScience) in the neck and the tail serving as reference and ground, respectively. Dark-adapted, full-field ERGs were recorded (Espion ColorDome ganzfeld, Diagnosys LLC, Lowell, MA) at a 1 kHz sampling rate and initially band-pass filtered at 0.3 to 300 Hz. Light stimuli consisted of 4 ms flashes at an intensity of 6.69 Cd⸱s/m^2^, an inter-flash interval of 10 seconds, and 3 traces recorded to obtain the average response [30]. Recordings were less than 30 minutes long.

ERG amplitudes and implicit times (IT) for the a-wave, b-wave, and oscillatory potentials (OPs) were calculated. After OPs were removed by filtering (75 Hz low pass), the a-wave amplitude was measured from baseline to the a-wave trough, and the b-wave amplitude was measured from a-wave trough to b-wave peak. IT was measured from stimulus onset to the respective trough/peak. OPs were extracted by bandpass filtering from 75 to 300 Hz [31]. Amplitudes and ITs for five individual OPs were measured, and the summed amplitude and IT for OPs were calculated and reported [30].

### Statistical analysis

Data are reported as mean ± standard error of the mean (SEM), with statistically significant differences reported for p < 0.05. ERG data were analyzed by averaging results from left and right eyes, and using t-tests to compare between O_2_-treated and untreated mice. Blood flow and IOP data were analyzed using linear mixed models using restricted maximum likelihood estimation, type III sums of squares, and Satterthwaite approximation of the degrees of freedom in SPSS 27 (IBM, Armonk, NY), and the results of F tests were reported. Treatment group (untreated or O_2_) and age were included in the model as fixed factors, and a random intercept for subjects was included. For blood flow measurements, values under baseline (breathing air) and hypercapnic conditions were also included with the breathing condition as a fixed factor, and a full factorial design was used. For IOP measurements, the eye from which the measurement was made was included as a fixed factor without interaction terms with the other factors. For post-hoc tests, unpaired t-tests were used to compare between treatment groups at the same age, paired t-tests were used to compare blood flow between baseline and hypercapnia, and a partially overlapping samples t-test was used to compare between ages (Partiallyoverlapping 2.0 package in R 4.0.3) [32]; Bonferroni-Holm correction for multiple comparisons (9 comparisons for IOP and 6 each for RBF and ChBF) was used with the unadjusted p-values reported.

## Results

**Fig 1:**
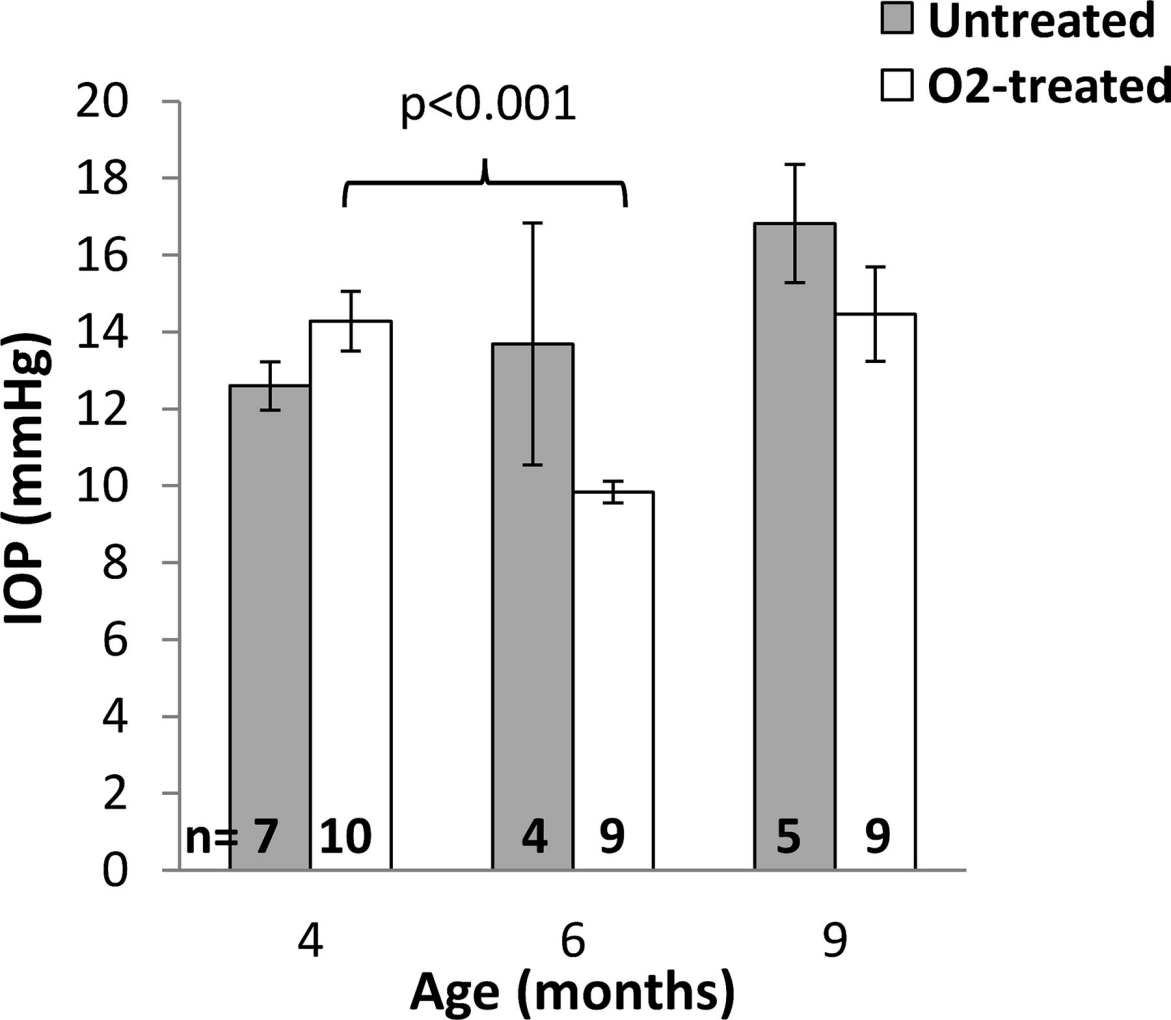
Intraocular pressure from DBA/2J mice. Mice were housed under room air (untreated) or 30% oxygen (O_2_-treated) beginning at 4.5 months of age. IOP was measured at 4, 6, and 9 months of age. Group means ± SEM are plotted after averaging the IOP of both eyes. The numbers of animals for each group are shown. Significant differences from post-hoc tests are indicated.

To determine whether decreased blood flow and hypoxia contribute to glaucoma, DBA/2J mice were given chronic mild hyperoxia treatment. IOP measurements were obtained from DBA/2J mice that were housed under room air or 30% oxygen beginning at 4.5 months of age (Fig 1). IOP from mice at 4, 6, and 9 months of age are given in Table 1. Significant effects of age (p < 0.001) and the age*treatment term (p = 0.028) on IOP were found, while O_2_-treatment did not have a significant effect (p = 0.14), indicating that the O_2_ altered how IOP changed with age compared to untreated animals. The left/right eye did not have a significant effect (p = 0.47), as would be expected. The IOP values of untreated DBA/2J mice progressively increased with age but were not significantly different between age groups from post-hoc tests. There were no significant differences between untreated and O_2_-treated mice at any time point. A significant decrease was observed in the IOP of O_2_-treated DBA/2J at 6 months compared to 4 months of age (p < 0.001), with IOP trending to then increase at 9 months (p = 0.010).

**Table 1:** IOP (average of left and right eyes) in untreated and 30% O_2_-treated DBA/2J mice (mean ± SEM).

	IOP (mmHg)		
	4 mo	6 mo	9 mo
Untreated	12.6 ± 0.6	13.7 ± 3.1	16.8 ± 1.5
O_2_-treated	14.3 ± 0.8	9.8 ± 0.3 ^a^	14.5 ± 1.2

^a^ p < 0.001, 6 mo vs. 4 mo

**Fig 2:**
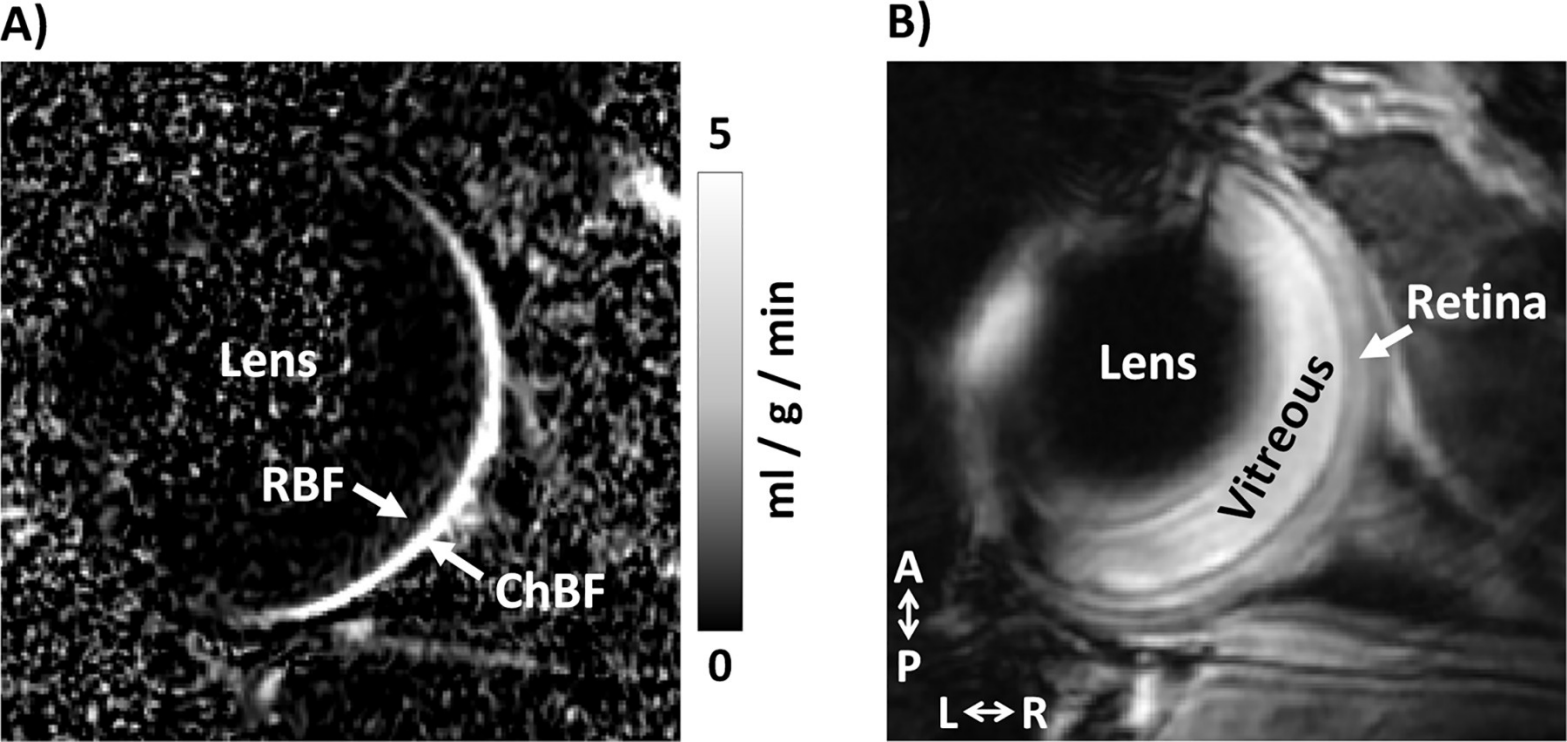
Blood flow MRI images of a mouse eye. **(A)** A blood flow map and **(B)** the corresponding anatomical EPI image. Retinal blood flow (RBF) and choroidal blood flow (ChBF) are indicated. A: anterior, P: posterior, L: left, R: right. The blood flow map is scaled from 0 to 5 ml/g/min.

**Fig 3:**
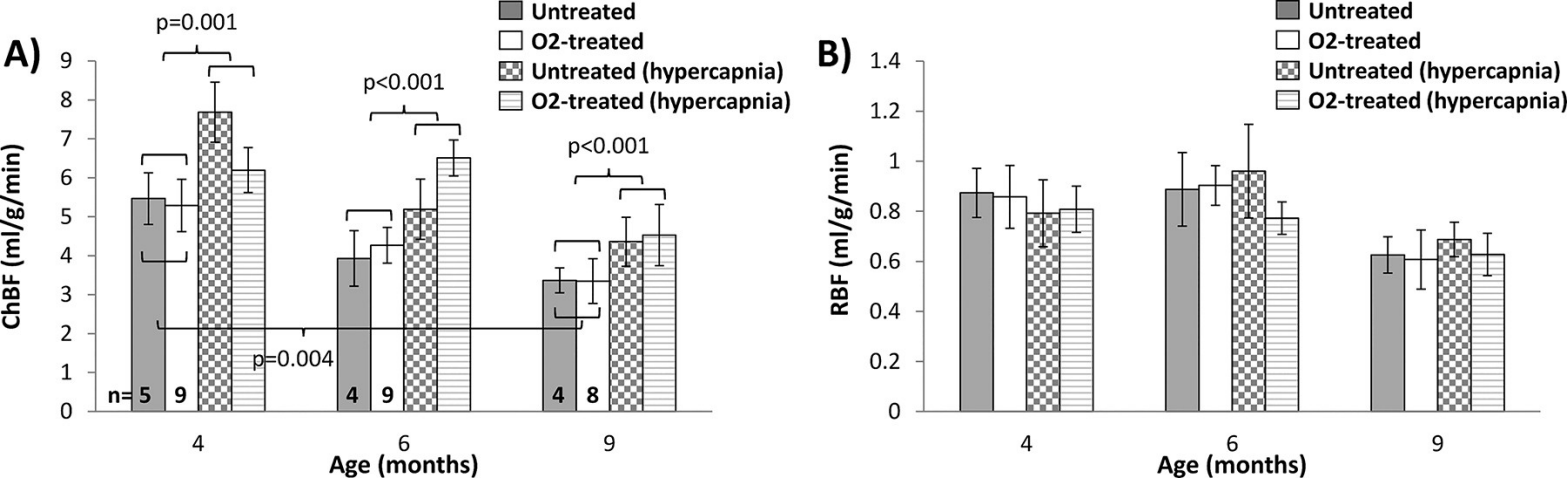
Group average ocular blood flow in DBA/2J mice. **(A)** Choroidal blood flow (ChBF) and **(B)** retinal blood flow (RBF) from DBA/2J mice housed under room air (untreated) or 30% oxygen (O_2_-treated) beginning at 4.5 months of age. Blood flow was measured at 4, 6, and 9 months of age. Basal blood flow measurements were made while the animals breathed room air and during hypercapnic challenge (breathing 5% CO_2_ in air). The numbers of animals for each group are shown in (A). Mean ± SEM. Significant differences from post-hoc tests are indicated.

Representative blood flow images are shown in Fig 2. Blood flow of the DBA/2J mice was measured under two conditions to assess basal blood flow and vascular reactivity: while the animals breathed room air and during hypercapnia challenge (air with 5% CO_2_). ChBF and RBF values are given in Table 2 and shown in Fig 3. ChBF (Fig 3A) progressively decreased with age in both O_2_-treated and untreated DBA/2J mice. ChBF was significantly affected by age (p < 0.001) and by hypercapnia (p < 0.001), while O_2_-treatment did not have a significant effect (p = 0.97). No interactions terms significantly affected ChBF (p > 0.2). For post-hoc tests, O_2_-treated and untreated groups were combined given the lack of differences. ChBF was significantly increased by hypercapnia at all ages (p = 0.001 at 4 months, p < 0.001 at 6 and 9 months). ChBF was significantly reduced at 9 months compared to 4 months (p = 0.004).

**Table 2:** Retinal and choroidal blood flow in untreated and 30% O_2_-treated DBA/2J mice (mean ± SEM).

		RBF (ml/g/min)			ChBF (ml/g/min)		
		4 mo	6 mo	9 mo	4 mo	6 mo	9 mo
**Basal**	Untreated	0.87 ± 0.10	0.89 ± 0.15	0.63 ± 0.07	5.47 ± 0.66	3.93 ± 0.71	3.37 ± 0.32 ^b^
	O_2_-treated	0.86 ± 0.13	0.90 ± 0.08	0.61 ± 0.12	5.29 ± 0.67	4.26 ± 0.46	3.35 ± 0.58 ^b^
**Hyper-capnia**	Untreated	0.79 ± 0.13	0.96 ± 0.19	0.69 ± 0.07	7.69 ± 0.77*	5.19 ± 0.77*	4.36 ± 0.63*
	O_2_-treated	0.81 ± 0.09	0.77 ± 0.06	0.63 ± 0.08	6.20 ± 0.58*	6.51 ± 0.46*	4.53 ± 0.78*

^b^ p < 0.005, 9 mo vs. 4 mo (combined untreated and O_2_-treated groups)

* p < 0.005, hypercapnia vs. basal (combined untreated and O_2_-treated groups)

Retinal blood flow values were measured for the O_2_-treated mice and untreated mice as well (Fig 3B**)**. RBF appeared similar between 4 and 6 months of age in both O_2_-treated and untreated groups and was lower at 9 months in both groups. RBF was significantly affected by age (p = 0.006) but not by hypercapnia (p = 0.77) or O_2_-treatment (p = 0.64). No interaction terms significantly affected RBF (p > 0.5). For post-hoc tests, O_2_-treated and untreated groups were combined given the lack of differences, which showed that RBF at 9 months trended to be lower compared to 4 months (p = 0.027) and 6 months (p = 0.026). Overall, the increased oxygen levels could lead to the regression of retinal blood vessels, which could be a potential problem with chronic O_2_ therapy. However, after 5 months of 30% O_2_-treatment, the basal blood flow and vascular reactivity of the choroidal and retinal vasculatures were not significantly affected when compared to untreated DBA/2J mice.

**Fig 4:**
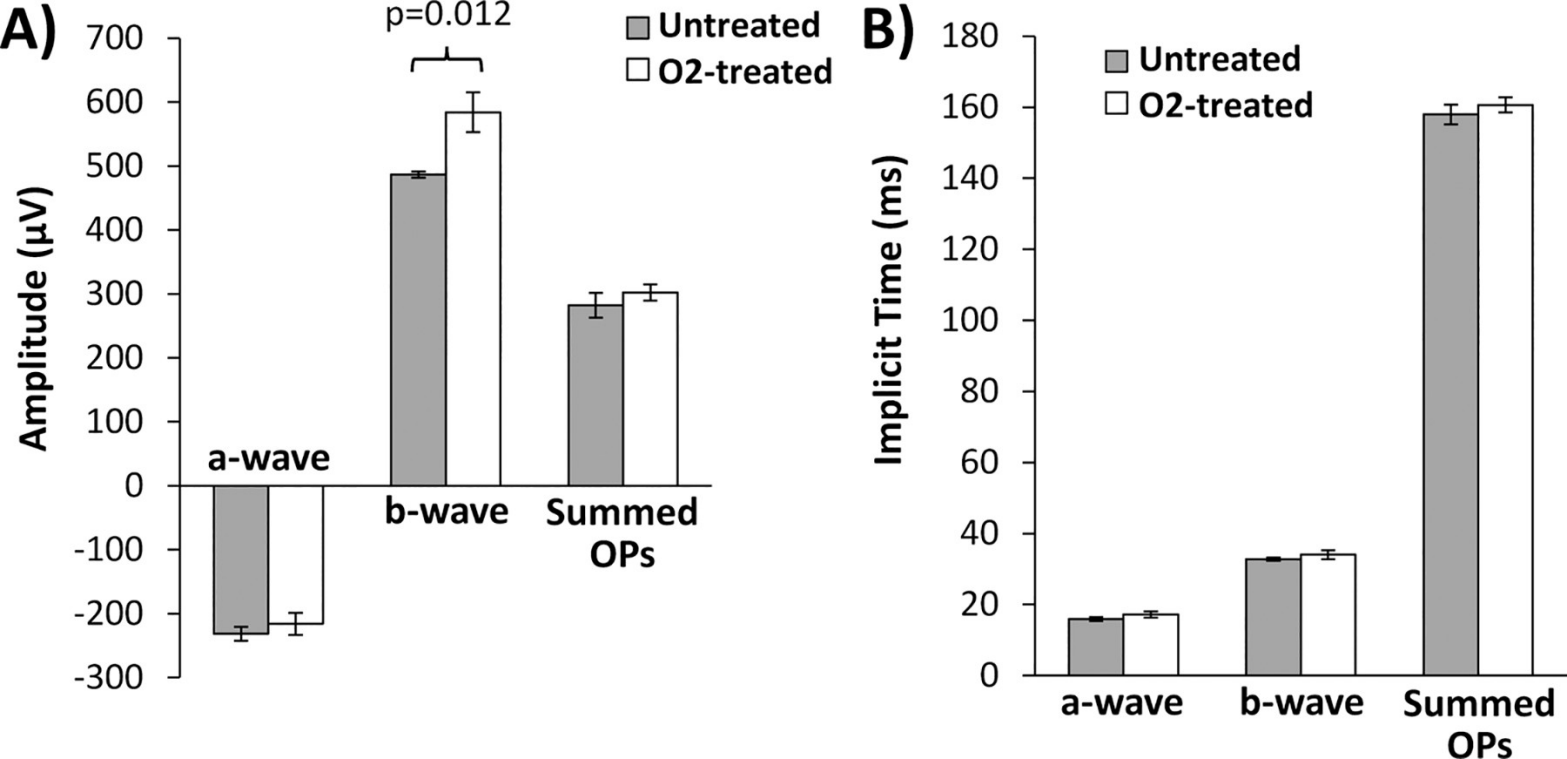
ERG from DBA/2J mice at 9 months of age. Mice were housed under room air (untreated, n = 5) or 30% oxygen (O_2_-treated, n = 10) beginning at 4.5 months. ERG data from both eyes were averaged and the **(A)** amplitudes and **(B)** implicit times of the a-wave, b-wave, and summed oscillatory potentials (OPs) are shown. Mean ± SEM. Significant differences are indicated.

From the flash ERG, the mean amplitudes (Fig 4A) and implicit times (Fig 4B) of the a-wave, b-wave, and summed oscillatory potentials (OPs) were determined (Table 3). The amplitude of the b-wave was significantly increased in O_2_-treated DBA/2J compared to the untreated mice (p = 0.012). The amplitudes of the a-wave and OPs were not significantly different between the O_2_-treated and untreated DBA/2J mice (p > 0.05). Implicit times of the a- and b-waves and of the OPs were not significantly different between the O_2_-treated and untreated mice (p > 0.05).

**Table 3:** ERG data (average of left and right eyes) from untreated and 30% O_2_-treated DBA/2J mice at 9 months of age (mean ± SEM).

	Amplitudes (μV)			Implicit times (ms)		
	a-wave	b-wave	OPs	a-wave	b-wave	OPs
Untreated	-231.6 ± 10.7	486.5 ± 4.9	282.0 ± 19.3	15.9 ± 0.5	32.8 ± 0.5	158.0 ± 2.8
O_2_-treated	-216.1 ± 17.3	584.0 ± 31.2 ^#^	302.4 ± 12.6	17.3 ± 0.9	34.1 ± 1.2	160.7 ± 2.1

^#^ p < 0.05, O_2_-treated vs. untreated

## Discussion

According to the ischemic hypothesis of glaucoma, ocular blood flow deficits may lead to neurodegeneration of retinal ganglion cells [5, 6, 20]. Currently, the only approved treatment for glaucoma is lowering IOP [1, 33]. Oxygen is important for the normal functioning of the retina [34–37]. Therefore, examining the role that oxygen has in the pathogenesis of glaucoma is important in understanding its ischemic mechanism. To test whether hypoxia due to impaired ocular blood flow contributes to glaucomatous progression, we measured blood flow and retinal function in DBA/2J mice treated with chronic, mild hyperoxia. RBF and ChBF declined with age in the DBA/2J mice and were not affected by the mild hyperoxia treatment. After 5 months of treatment with hyperoxia, the ERG b-wave amplitude was significantly increased compared to untreated mice. These studies provide evidence supporting that hypoxic ischemia could be a pathogenic factor in glaucoma and that treatments targeting hypoxia could potentially be beneficial in glaucoma.

Reduced ocular blood flow in glaucoma has been reported in animal models and human patients. In our previous studies, we showed that blood flow in the retinal and choroidal circulations decrease significantly as a function of age in DBA/2J mice [10, 18], consistent with our findings herein. In glaucoma patients, decreased ocular blood flow [5] and decreased retinal and optic disc vascular density [38] have been reported. In addition to basal blood flow, we also assessed vascular reactivity using hypercapnic inhalation which causes vasodilation. The ChBF increased in response to hypercapnia, but the response was not significantly affected by age, suggesting that the choroidal vascular responsiveness remained relatively normal despite reduced basal blood flow. Hypercapnia did not significantly affect RBF herein, while previous studies have reported increased RBF in response to hypercapnia using microspheres in animals [39] or increased retinal blood velocity or retinal vessel dilation in people [40, 41], although vessel diameter is also reported not to change [40]. One possible reason for this discrepancy is that the RBF MRI signal is low compared to the ChBF, so more animals or longer scans may be needed to detect a significant RBF response to hypercapnia. It may also be that anesthesia blunted the RBF response or that the retinal vasoreactivity was impaired in the DBA/2J mice even at 4 months of age.

Increased oxygen levels could lead to the regression of retinal blood vessels, which could be a potential problem with chronic oxygen therapy. The retinal vasculature is sensitive to high levels of oxygen and may regress under long term hyperoxia [22, 42], particularly during development as in oxygen induced retinopathy [43]. However, five months of the mild O_2_-treatment did not significantly affect basal ChBF and RBF or their responses to hypercapnia, so the 30% O_2_ level was likely low enough to avoid impairing the retinal and choroidal blood vessels.

The DBA/2J mouse has well-documented increases in IOP as a function of age [16, 44–46]. There were significant differences in the changes of IOP with age in the O_2_-treated and untreated mice. The O_2_-treated mice had a transient decrease of IOP at 6 months of age compared to 4 months. In the untreated group IOP trended to progressively increase with age as would be expected. Acute normobaric and hyperbaric oxygen have been reported to reduce IOP [47–49], which returned to normal post-treatment [49]. However, acute hyperoxia combined with hypercapnia has also been reported to not affect IOP [50], and repeated hyperbaric oxygen treatments did not affect IOP following the treatment [51, 52]. In contrast, increased aqueous and vitreous oxygen has also been associated with the development of glaucoma, thought to be due to increased reactive oxygen species which damage the trabecular meshwork, impeding aqueous outflow [53]. One limitation of this study is that the IOP spans a wide range in DBA/2J mice and measurements can be impacted by corneal calcification [44, 46, 54], so further investigations with larger groups and with invasive IOP measurements are warranted to better understand the effects of mild, chronic hyperoxia on IOP in glaucoma.

Previous studies have shown ERG abnormalities in DBA/2J mice are associated with ocular hypertension and age-related development of glaucoma [16, 21, 55, 56]. The scotopic a- and b-wave amplitudes progressively decline by around 8 months of age in DBA/2J and DBA/2NNia mice compared to normal C57BL/6J mice [55, 57]. The implicit times of the b-wave [55, 57] and a-wave [55] have also been reported to increase with age in DBA mice. The b-wave is associated with synaptic activity and inner neural layer of the retina, including bipolar and Müller cells. In retinal ischemia models, the b-wave has been documented to be more sensitive than the a-wave [21, 58]. This helps explain the significant improvement of the b-wave amplitude between O_2_-treated and untreated DBA/2J mice at 9 months. Chronic, high-levels of hyperoxia (e.g. 100% breathing) can also cause photoreceptor degeneration after a few days to weeks [22], but the ERG a-wave was not adversely affected herein, so mild 30% oxygen avoided potential oxygen toxicity.

Hyperoxia should increase retinal oxygenation which could compensate for potential hypoxia due to the reduced ocular blood flow. A few studies have shown increased expression of hypoxia inducible factor (HIF-1) in the retina of glaucoma patients [23, 59], supporting that hypoxia can occur in the glaucomatous retina. In a rat induced-model of ocular hypertension, erythropoietin, which may be protective against hypoxic injury, was shown to reduce HIF-1 expression and to prevent deficits in the ERG b-wave [60], consistent with our results with O_2_-treatment. Alternatively, the O_2_-treated mice had reduced IOP at 6 months of age, so the IOP-reduction could have also contributed to the improved ERG function. In human diabetic patients, 100% oxygen treatment given acutely improved contrast sensitivity deficits and improved the amplitude of the ERG OPs [61, 62]. Diabetic retinopathy involves several vascular abnormalities leading to ischemia and hypoxia in the retina [63], which suggests that hyperoxia may be beneficial in retinal diseases associated with impaired blood flow. However, in contrast to these studies, our mice were chronically treated with oxygen but were studied under normal air after 24-hr acclimation, so further studies are needed to explore acute versus chronic effects. This result suggests that chronic treatment provides a prolonged improvement, but how long retinal function may remain improved after discontinuation of treatment remains to be explored. Regarding the feasibility for administering such chronic treatment, further studies will be required to examine the optimal treatment length and concentration for glaucoma models, such as whether a few hours of 100% O_2_ per day could give similar protection as continuous 30% O_2_. We found that chronic hyperoxia at 30% O_2_ for 5 months did not affect ChBF and RBF, suggesting that this level did not have adverse effects and was relatively safe, but histochemical studies of vascular and neuronal health could further confirm this. Chronic treatment for humans may potentially be administered by facemask depending on whether repeated short periods of treatment given chronically have similar effects as continuous treatment, although it should be noted that such treatments would only serve as supplemental to treatments that target IOP. IOP decreased significantly at 6 months and its significance disappeared at 9 months, while the retinal function was improved at 9 months, suggesting chronic treatments can provide long-term improvements. The present study adds to the existing literature by showing the effects that chronic, mild hyperoxia treatment has on retinal blood flow and function in the DBA/2J mouse model of glaucoma.

## Conclusions

This study investigated the effects of chronic mild hyperoxia (30% O_2_) on retinal function and on retinal and choroidal blood flow in a mouse model of glaucoma. Retinal function from ERG was improved in the O_2_-treated mice at late stage, despite a progressive decline of RBF and ChBF with age which was comparable to untreated mice. The results suggest that oxygen treatment could provide an alternative treatment strategy for glaucoma in addition to standard IOP-lowering strategies. Future studies should further investigate the relationship between blood flow, oxygenation, and IOP with glaucomatous damage and vision loss to elucidate the mechanisms of ischemia and hypoxia in glaucoma.

## Supporting information

S1 Table Data from individual subjects. All IOP, blood flow, and ERG data are given.
(XLSX)
